# Study of PVP and PLA Systems and Fibers Obtained by Solution Blow Spinning for Chlorhexidine Release

**DOI:** 10.3390/polym17131839

**Published:** 2025-06-30

**Authors:** Oliver Rosas, Manuel Acevedo, Itziar Vélaz

**Affiliations:** 1 Department of Chemistry, Faculty of Science, University of Navarra, 31080 Pamplona, Spain; oarosas@unav.es; 2 Department of Sciences and Engineering, Iberoamerican Puebla University, Puebla 71820, Mexico; manuel.acevedo@iberopuebla.mx

**Keywords:** polymeric fibers, solid dispersions, chlorhexidine, Solution Blow Spinning, drug delivery

## Abstract

Antimicrobial resistance arises from treatment non-adherence and ineffective delivery systems. Optimal wound dressings combine localized drug release, exudate management, and bacterial encapsulation through hydrogel-forming nanofibers for enhanced therapy. In this work, polylactic acid (PLA) and polyvinylpyrrolidone (PVP) fibers loaded with chlorhexidine (CHX) were developed using Solution Blow Spinning (SBS), a scalable electrospinning alternative that enables in situ deposition. Molecular interactions between CHX and polymers in solution (by UV-Vis and fluorescence spectroscopy) and in solid state (by FTIR, XRD and thermal analysis) were studied. The morphology of the polymeric fibers was determined by optical microscopy, showing that PVP fibers are thinner (1625 nm) and more uniform than those of PLA (2237 nm). Finally, drug release from single-polymer fibers discs, overlapping fibers discs (PLA/PVP/PLA and PVP/PLA/PVP), and solid dispersions was determined by UV-Vis spectrometry. PVP-based fibers exhibited faster CHX release due to their hydrophilic nature, while PLA fibers proved sustained release, attributed to their hydrophobic matrix. This study highlights the potential of PLA/PVP-CHX fibers made from SBS as advanced wound dressings, combining biocompatibility and personalized drug delivery, offering a promising platform for localized and controlled antibiotic delivery.

## 1. Introduction

The dramatic increase in antibiotic resistance among pathogenic microorganisms has become a critical global health concern, affecting not only the number of resistant strains but also their geographical distribution and resistance levels [1]. The dissemination of microorganisms is increasing quickly, posing a global public health problem [2,3]. This is mainly due to lack of adherence to treatment by patients, especially in countries where antimicrobials are readily available over the counter. Notably, pathogens such as *E. coli*, *P. aeruginosa* and *A. baumannii* have emerged as significant threats [4].

Conventional immediate-release delivery systems are not able to provide optimal therapy for chronic diseases and can lead to adverse effects. The rapid, fluctuating drug concentrations they produce are insufficient for maintaining therapeutic levels. In consideration of this, it is essential to develop materials that improve the drug bioavailability and efficacy, such as polymeric nanofiber-based transdermal patches [5].

In the treatment of wounds, the ideal dressing should be easy to apply, promote rapid healing, and be cost-effective. Modern dressings seek optimal conditions for epithelial regeneration, including maintaining a moist environment, ensuring proper oxygenation, and minimizing bacterial load. The choice of dressing depends on the wound type (whether acute, chronic, exudative, or dry) and its depth [6]. Current commercials dressings, such as Protec^®^ (Degasa, Mexico), Hyalofast^®^ (Anika, USA), Hydrofilm^®^ (Hartmann, Germany) and Aquacel^®^ (Convatec, UK), are typically made from microfibers, which have limited surface area for mass transfer and cannot prevent bacterial invasion as effectively as nanofibers [7]. Nanofibrous materials in dressings can absorb wound exudates and, depending on their composition, may swell to form hydrogels that trap bacteria [6].

Polymeric materials are widely used in biomedicine due to their versatile properties. Biodegradable polymers like polylactic acid (PLA), polyvinyl alcohol (PVA), polycaprolactone (PCL), hydrogels, collagen, chitosan, and hyaluronic acid are commonly employed in wound dressing and drug delivery systems [6]. Their effective uses have been reported in tissue engineering [8,9,10], drug delivery [11,12,13], personalized cell therapy [14], and cancer treatment by immunotherapy [15]. For instance, Farkas et al. demonstrate that the drug release of doxycycline from PLA nanofibers was controlled by Fick diffusion processes, best fitting the Korsmeyer–Peppas model [16].

Polyvinylpyrrolidone (PVP) is particularly notable for its stability, hygroscopicity, surface activity, and solubility in various solvents, owing to its hydrophilic and hydrophobic functional groups. Its biocompatibility and ability to form stable complexes with active substances make it valuable in pharmaceutical formulations, including tablets, hydrogels and coatings for medical devices, among others [14]. This polymer is used for its antioxidant properties in dressings containing drugs to treat wound pain and inflammation. Razzak et al. have shown that optimized PVA and PVP hydrogels meet key requirements for wound dressings such as fluid absorption, painless removal, and elasticity [17]. Additionally, PVP has been combined with natural extracts like *Isatis* root to produce antimicrobial nanofibers by electrospinning [18], emphasizing its versatility [19].

PLA is a linear aliphatic thermoplastic polyester, composed of lactic acid monomers, is biodegradable and serves as a sustainable alternative to conventional polymers in packaging and insulation [20]. PLA is approved by the European Medicines Agency (EMA) for medical applications such as degradable sutures, stents and wound dressings. The intrinsic porosity of PLA confers significant advantages for a broad range of biomedical applications including tissue engineering, controlled drug delivery, cancer therapeutics (e.g., tumor targeting and photothermal therapy), dental implants, and orthopedic devices [21,22]. Recent work by Liang et al. highlights the potential of PLA-lignin nanofibers as scaffolds that promote cartilage regeneration, offering a promising approach for osteoarthritis management [23]. Moreover, quercetin-loaded PLA fibers have shown efficacy as pH-responsive drug carriers, particularly for the localized treatment of periodontitis [24].

The Solution Blow Spinning (SBS) technique has emerged as a fast and scalable method for producing polymeric and ceramic nanofibers with diameters below 100 nm using pressurized gas. Unlike electrospinning (ES), SBS does not require an electric field, making it more versatile for in situ applications, such as direct deposition onto wound surfaces. While ES produces highly homogeneous fibers, its limitations include incompatibility with certain polymers and the inability to work on all substrates [25]. Recent studies have demonstrated SBS’s effectiveness produce nanomaterials, such as fibrous scaffolds [26] and micro- and nanofibers [27,28]. For example, Domínguez et al. showed that SBS could produce polysulfone materials with controlled morphology, with fiber size and quantity influenced by feeding rate, air pressure, and working distance [29].

**Figure 1:**
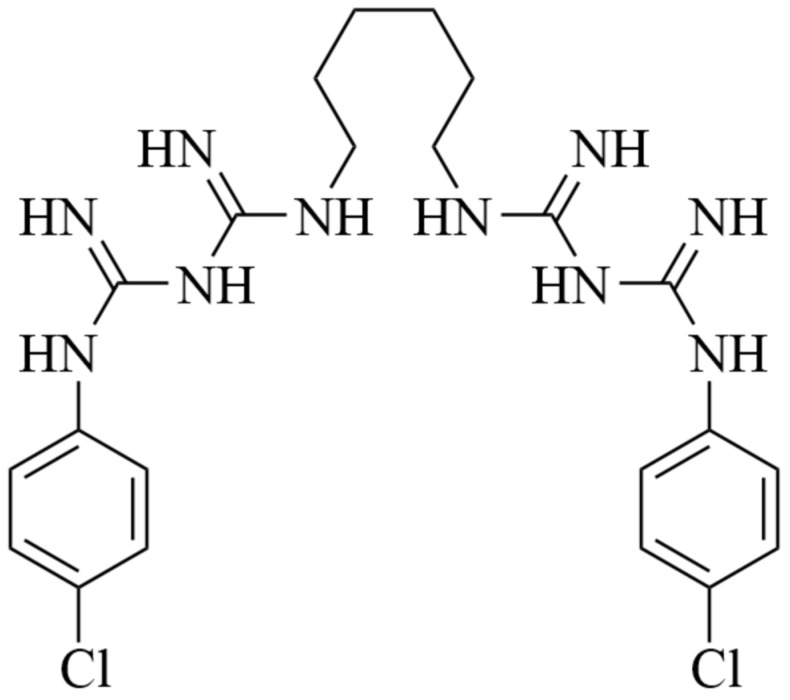
Chemical structure of chlorhexidine.

Chlorhexidine (CHX) is a potent antiseptic used to prevent or treat burn would infections and reduce polymicrobial biofilms formation [30,31]. CHX is active against Gram-positive and Gram-negative bacteria, yeasts and viruses; it is bacteriostatic at low concentrations (0.02–0.06%) and bactericidal at higher concentrations (>0.12%) [32]. CHX consists of two cationic biguanide groups attached to a 4-chlorophenyl ring and linked together by a hexamethylene chain (Figure 1). These cationic groups are responsible for the pharmacological activity; the phosphate groups bind to microbial cell walls and membranes, causing coagulation and precipitation of bacteria until cell death [33].

Recent research has explored CHX incorporation intro polymeric fibers by ES. For example, de Carvalho et al. developed cellulose acetate and polyethylene oxide (PEO) nanofibers loaded with CHX for dental applications, highlighted an effective encapsulation and controlled release of CHX [34]. Other studies, like Bardonova et al. produced hydrophobic polymer fibers with CHX and kaolinite, achieving very slow-release rates (5% over 168 h) [35]. Additionally, CHX-loaded membranes for dental implants have been developed to prevent biofilm formation [36,37]. Despite these advances, a critical gap remains in the application of SBS for fabricating antimicrobial wound dressings containing chlorhexidine (CHX), particularly using optimized PLA/PVP blends.

Owing to its excellent biocompatibility and biodegradability, PLA is extensively employed in the development of skin and wound dressing. Nonetheless, its inherent hydrophobicity can compromise cytocompatibility and limit biological performance. To overcome these limitations, PLA is often blended with hydrophilic polymers such as PVP, enhancing its wettability and overall biocompatibility [38]. Recent advances in polymer science have identified promising approaches to optimize CHX delivery. Bonan et al. demonstrated that PLA/PVP blends produced via SBS could effectively encapsulate copaiba oil [39], while Zhou et al. have shown that electrospun PLA/PVP fibers enable efficient salicylic acid release, while also exhibiting notable antibacterial properties [38], and Scaffaro et al. showed that graphene nanoplatelets in PLA matrices could slow CHX release [40]. However, no studies have systematically investigated the molecular interactions between CHX and PLA/PVP blends, nor optimized SBS parameters for CHX-loaded fibers and neither analyzed the kinetic parameters and drug release profiles from solid dispersions. This represents a critical gap in the field, as understanding these interactions is essential for controlling release kinetics and ensuring antimicrobial efficacy.

This study aims to develop and characterize PLA and PVP fibers loaded with CHX, fabricated using the SBS technique, as well as to analyze the kinetic parameters and drug release profiles from solid dispersions and fibers. These polymers, due to their biocompatibility, highly porous structure and morphological diversity in the form of fibers obtained by SBS could provide significant advantages, such as a direct application on the affected area, promoting the absorption and release of the antibiotic in a controlled manner.

## 2. Materials and Methods

### 2.1. Material

Polyvinylpyrrolidone (PVP, M_w_ = 360,000 g/mol, Sigma-Aldrich), polylactic acid (PLA, M_w_ = 35,000 Da; produced by Resinex Spain, SL, and manufactured by Nature Works LLC, Blair, NE, USA), chlorhexidine (CHX, ≥99.5%, M_w_ = 505.45 g/mol, Sigma-Aldrich), ethanol (99.5%, PanReac AppliChem) and dichloromethane (DCM, 99.95%, Quimipur).

### 2.2. Molecular Interactions in Solution

#### 2.2.1. Ultraviolet Visible Spectroscopy (UV-Vis)

Dissolutions of 1.0 × 10^−5^ M of CHX in 6 and 10% *w*/*w* PVP and 6 and 8% *w*/*w* PLA were prepared using ethanol and dichloromethane as solvent, respectively. Absorbance spectra measured over a range of 200 to 325 nm were obtained using an Agilent Cary 8454 diode array UV-Visible spectrophotometer (Santa Clara, CA, USA).

#### 2.2.2. Fluorescence Spectroscopy

1.0 × 10^−6^ M dilutions of CHX in 9% *w*/*w* PVP and PLA were prepared using ethanol and dichloromethane as solvent, respectively. Fluorescence emission spectra were collected over a range of 300 to 400 nm (PVP) and 300 to 440 nm (PLA) by means of a Perkin Elmer LS-50B spectrofluorometer; λ_exc_: 290 nm, slit: 3 nm performing 5 repetitions per assay.

### 2.3. Molecular Interactions in Solid State

#### 2.3.1. Preparation and Characterization of Solid Dispersions (SD)

Solid dispersions of CHX-PVP and CHX-PLA were prepared using the cosolvent method [41] by dissolving the polymers and drug separately using the minimum amount of solvents (ethanol for PVP and dichloromethane for PLA) at drug/polymer ratios 30–70, 40–60 and 50–50 *w*/*w*. Once dissolved, the drug was mixed with each polymer and the solvent was removed by rotavapor (Rotavapor R-200, vacuum controller V-800 and heating bath B-490 Büchi, bath temperature 100 °C and pressure 100 mbar) for ethanol, and by natural evaporation for dichloromethane. For comparative purposes, physical mixtures (PM) of CHX-PVP and CHX-PLA in 50-50 *w*/*w* ratio were prepared, which consisted of mixing the drug with each polymer in solid state.

#### 2.3.2. Fourier Transform Infrared Spectroscopy (FTIR)

Once the solvent was removed from the aforementioned dispersions, their FTIR spectra were obtained, as were those of CHX, PVP, and PLA in the solid state using a Shimadzu IRAffinity-1S FTIR spectrometer equipped with a diamond ATR attachment, and the spectra were recorded at a resolution of 4 cm^−1^ (100 scans).

#### 2.3.3. X-Ray Diffraction (XRD)

Solid dispersions were analyzed over a range of 5° to 60° (2Ө), D (2Ө) of 0.03° per step and a step speed of 2 s. A Bruker D8 X-ray diffractometer and a Kristalloflex K760 X-ray generator were used.

#### 2.3.4. Thermogravimetric Analysis (TGA)

The analyses were performed from 35–1000 °C with a heating rate of 20 °C/min. The equipment used was a TA Instruments SDT650.

#### 2.3.5. Differential Scanning Calorimetry (DSC)

The calorimetry spectra of the solid dispersions were analyzed. Measurements were collected from −20 to 200 °C with a step rate of 20 °C/min using a TA Instruments DSC25 calorimeter.

### 2.4. Fiber Processing

Solutions of PVP and PLA 6% *w*/*v* dissolved in ethanol and dichloromethane, respectively, were prepared, to which 0.46% *w*/*w* chlorhexidine was incorporated with respect to the polymer on a dry basis; for this purpose, 3 g of polymer were dissolved in 50 mL of solvent and 13.8 mg of drug were added. Once the solutions were dissolved, they were placed in an SBS equipment for obtaining fibers, consisting of a spinning system (XTech 500 professional airbrush, SAGOLA, with a 0.5 mm concentric nozzle), a mesh collector with an object holder and a compressed air source (Wenzhou Hanfong Machinery Co., Ltd., 3 L, 1–5 HP). The solution was injected through the internal nozzle with a flow rate of 129.31 µL/min and pressurized gas was supplied with a pressure of 1.4 ± 0.2 bar.

#### 2.4.1. Morphological Analysis

Fiber samples were analyzed on an Axiolab 5 optical microscope (Carl Zeiss Suzhou, Co., Ltd., Suzhou, China) at magnifications of ×20, ×50 and ×100. Fiber diameter was determined using ImageJ software (Java Version 1.54b) by making 50 measurements per sample image. The collected data were analyzed using OriginPro 2019b software (OriginLab Version 9.6.5.169) to determine the average diameter and distribution of fibers.

#### 2.4.2. Chlorhexidine Release Assays from Solid Dispersions and Fibers

Release assays from solid dispersions were developed using the USP basket method at 50 rpm [42]. Samples were sieved between 160 and 250 μm particle size. The amount of each solid dispersion and PM prepared containing 7 mg of CHX was placed in a SOTAX AT7 smart vessel (Sotax, Basel, Switzerland) containing 500 mL of deionized water as release medium at 35.0 ± 0.2 °C. Aliquots of 2 mL were collected and replaced with fresh medium to maintain a constant volume.

In the case of fibers, to obtain homogeneous and reproducible assays, discs of 1.3 mm diameter and 1 mm width were made. For this purpose, 110 mg of fibers were weighed with drug and placed in a PerkinElmer 25T hydraulic press with a pressure of 5 × 10^−4^ N for 5 min. On the other hand, mixed systems of drug discs were prepared with the two polymers. 110 mg of total fibers were placed in the press in the following order: 40% *w*/*w* PLA, 20% *w*/*w* PVP and 40% *w*/*w* PLA with respect to the total weight to obtain PLA/PVP/PLA mixed discs. Mixed PVP/PLA/PVP fiber discs were also prepared. Fiber assays were performed by manual sampling by placing the fiber-based discs in baskets inside beakers with 20 mL of release medium at room temperature and 800 µL aliquots were collected. Assays were performed in triplicate.

To determine the amount of drug released during kinetics, the absorbance of the obtained aliquots was measured using a UV-Visible spectrophotometer (Cary 8454 UV-Vis, Agilent); absorbance was determined at the maximum absorption of the drug (λ_max_ = 254 nm) [43]. The following calibration line equation was used to determine the concentration of CHX released:

(1) $$y=28,709x+5.1\times 10^{-3}$$

where y is the absorbance obtained in the λmax and *x* is the [CHX] in molarity (M). Once the [CHX] was known, the percentage of chlorhexidine released in each sample with respect to the total amount of drug released was determined.

### 2.5. Mathematical Models

To elucidate the mechanisms of chlorhexidine release from SD and fibers, the results corresponding to 80% of chlorhexidine released with respect to the total amount of drug released in each experiment over time were fitted to six mathematical models: Korsmeyer–Peppas, Higuchi, order 0, order 1, Makoid–Banakar and Peppas–Sahlin [44,45,46,47,48,49,50].

Through the adjustments the value of the kinetic constant (k) was obtained in each case, the value of n of Korsmeyer–Peppas indicates the release mechanism of the drug and varies depending on the geometry of the release device. Values of n between 0.85 and 1 indicate non-Fickian swelling controlled release [48]; values around 0.45 correspond to a Fickian diffusion mechanism and, finally, with values of n > 1.00 the release process is known as Super Case II transport [46].

The Makoid–Banakar model is an empirical model that assumes that the release is a result of the sum of several mechanisms such as zero order whose constant is *k_MB_* and subsequently order 1 whose constant is represented by c, which are empirical parameters along with n:

(2) $$M_{t}/M_{\infty }=k_{MB}t^{n}exp(-ct)$$

where *t* is the time release (min), M_t_ is the amount of drug released as a function of time, and M_∞_ is the total amount of drug released [44,47,50].

In the Peppas–Sahlin model, the first term (*k_D_t^m^*) represents the diffusion contribution, where k_D_ is the diffusional constant and *m* is the diffusion exponent for a release system of any geometry. The second term (*k_R_t^2m^*) corresponds to the relaxation release contribution of the polymer chains where k_R_ is the relaxation constant [44,45,46,47,49,50]:

(3) $$M_{t}/M_{\infty }=k_{D}t^{m}+k_{R}t^{2m}$$

where *t* is the time release (min), M_t_ is the amount of drug released as a function of time, and M_∞_ is the total amount of drug released.

In all cases, the values of R^2^ and adjusted R^2^ were determined, since, as they indicate, the latter is more appropriate for choosing the best fit when comparing models with different numbers of parameters, as is the case here.

## 3. Results and Discussion

### 3.1. Molecular Interactions in Solution

To study the interactions in solution between the drug and the polymers PVP and PLA, mixtures of both were measured by UV-Vis and fluorescence spectroscopy.

#### 3.1.1. Ultraviolet Visible Spectroscopy (UV-Vis)

**Figure 2:**
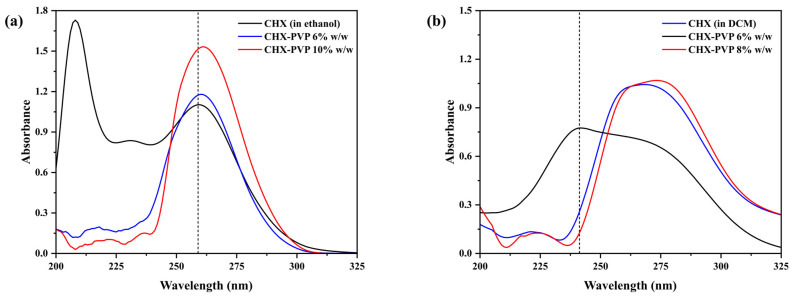
UV-Vis spectra of pure CHX at (**a**) 2.0 × 10^−5^ M and in presence of 6% *w*/*w* and 10% *w*/*w* PVP in ethanol and (**b**) 3.0 × 10^−5^ M and in presence of 6% *w*/*w* and 8% *w*/*w* PLA in dichloromethane.

Figure 2 shows the absorption spectra of pure chlorhexidine and in contact with PVP and PLA at different concentrations. The spectra of the CHX-PVP solutions show their λ_max_ at 259 nm and those of CHX-PLA at 267 nm. The observed differences in the CHX spectra may be due to the influence of the polarity of the solvent, ethanol or dichloromethane, depending on the polymer used. Skoog et al. indicate that polar solvents tend to erase the fine structure of the spectrum caused by vibrational changes, resulting in a uniform absorption band, whereas in non-polar solvents the electronic transitions can be observed, but the vibrational and rotational structure is lost [51]. The spectra show an increase in the absorbance of drug–polymer solutions, as well as a shift in the wavelength where the maximum absorbance is found as a function of the concentration of the polymer used. These changes are more evident the higher the concentration of the polymer used, being more noticeable in the CHX-PLA solutions. This suggests the presence of molecular interactions between chlorhexidine and each of the polymers [52,53].

#### 3.1.2. Fluorescence Spectroscopy

**Figure 3:**
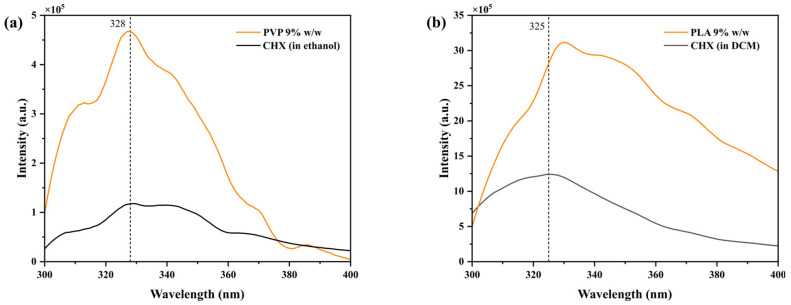
Emission spectra of CHX 1.0 × 10^−6^ M in the presence of 9% *w*/*w* (**a**) PVP and (**b**) PLA in ethanol and dichloromethane, respectively (λ_exc_: 290 nm).

The emission spectra of 1.0 × 10^−6^ M CHX solutions with 9% *w*/*w* PVP and PLA in ethanol and dichloromethane are plotted in Figure 3, respectively. The spectra show a maximum in the 320–340 nm range, corresponding to the chlorobenzenes present in this compound (275–345 nm) (Figure 1) [53]. Changes were observed in the maximum emission of the drug as well as in the drug intensity in the presence of the polymers, probably indicative of molecular interactions between the drug and the polymers [54].

### 3.2. Molecular Interactions in Solid State

#### 3.2.1. Fourier Transform Infrared Spectroscopy (FTIR)

**Figure 4:**
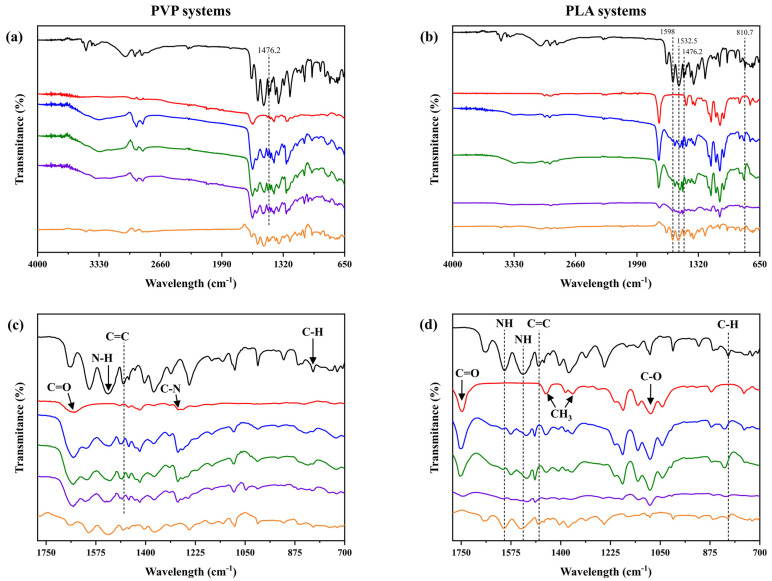
ATR-FTIR spectra of (**a**,**b**) full spectral range and (**c**,**d**) zoomed-in region (1780–700 cm^−1^) for CHX, polymers, CHX–polymer solid dispersions (30-70, 40-60 and 50-50), and physical mixtures (PM) 50-50 of CHX-PVP and CHX-PLA, respectively. Panels (**a**,**c**) correspond to CHX-PVP, while panels (**b**,**d**) correspond to CHX-PLA. Spectra are shown from top to bottom in the order listed.

The FTIR spectra of pure compounds and solid dispersions of CHX-PVP and CHX-PLA are shown in Figure 4. The FTIR spectra of chlorhexidine show main absorption peaks at 1598.0 cm^−1^ and 1532.5 cm^−1^ (N-H bending vibration of secondary amine and imine group), 1476.2 cm^−1^ (C=C stretching vibration of the aromatic ring) and 810.7 cm^−1^ (C-H bending vibration of aromatic ring) [55]. The PVP spectrum (Figure 4c) shows peaks at 1650.4 cm^−1^ (C=O bond stress mode of a δ-lactam) and 1284.5 cm^−1^ (band associated with the C-N stress mode of an amine) [56].

The spectra of the solid dispersions prepared by the cosolvent method present shifts in bands typical of chlorhexidine and PVP as is the case of the C=C bond (going from 1476.2 to 1486.5 cm^−1^). The physical mixture (PM) of these compounds is also depicted, where the typical peaks present in CHX and PVP are shown in the FTIR spectrum of this mixture. The FTIR spectrum of PLA (Figure 4d) shows transmittance peaks at 1749.5 cm^−1^ (C=O bond stress mode belonging to an ester group), 1450.9 cm^−1^ (CH_3_ bending band symmetric), 1358.9 cm^−1^ (CH_3_ bending band asymmetric), and 1087.6 cm^−1^ (C-O stress band of aliphatic ester) [57].

The FTIR spectra of the CHX-PLA solid dispersions (Figure 4b) show shifts in bands typical of chlorhexidine, such as N-H bonds (shifting from 1598.0 to 1575.9 cm^−1^ and from 1532.5 to 1520.1 cm^−1^), C=C (from 1476.2 to 1492.4 cm^−1^), and the addition of the C-H bond peak (shifting from 810.7 to 823.0 cm^−1^). The physical mixture (PM) spectrum is also presented, where typical CHX and PLA peaks are identified, as expected. The above suggests the presence of weak molecular interactions between chlorhexidine and each of the polymers in the cosolvent solid dispersions. In CHX-PVP systems, potential interactions appear to involve the carbonyl groups, which are generally prone to spectral shifts; however, only minimal displacement is observed in this region, especially when compared to the more prominent shift associated with the C=C bond. In contrast, CHX-PLA dispersions strongly suggest hydrogen bonding interactions, most likely occurring between the amine (N-H) groups of chlorhexidine (indicated by shifts at 1575.9 and 1520.1 cm^−1^) and the carbonyl (C=O) groups of PLA. Moreover, the lack of molecular interactions in the physical drug–polymer mixtures is proposed [40,58,59].

#### 3.2.2. X-Ray Diffraction (XRD)

**Figure 5:**
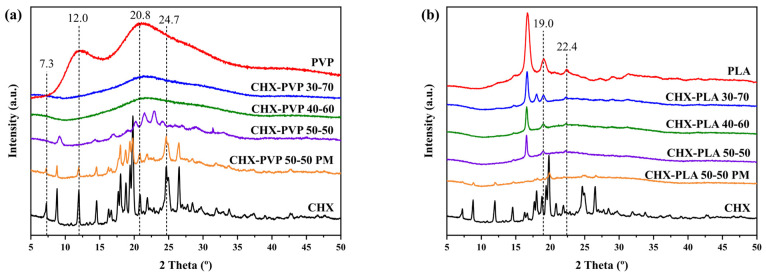
X-ray diffractograms of pure compounds, solid dispersions, and physical mixtures (PM) of (**a**) CHX-PVP and (**b**) CHX-PLA.

The diffractograms obtained for solid drug–polymer dispersions are shown in Figure 5. The XRD pattern of chlorhexidine has many peaks throughout the spectrum, showing highly crystalline patterns [60]. The spectrum of PVP (Figure 5a) shows two characteristic broad peaks at 2θ at 12.0° and 20.8° [61]. The diffractograms of the CHX-PVP solid dispersions show amorphous compounds at drug–polymer ratios 30-70 and 40-60. On the other hand, the CHX-PVP 50-50 composition shows changes in intensity and shift in the 2θ positions at 7.3°, 24.7°, 12.0°, and 20.8°, the latter two present in the diffractograms of the pure CHX and PVP compounds. The spectrum of the physical mixture is also represented, where typical peaks of both compounds are identified. The diffractogram of PLA (Figure 5b) presents two main peaks at 2θ at 16.7° and 19.1° [62]. The diffractograms of the solid dispersions show greater similarity with pure PLA; however, changes in intensity and position of 2θ are presented at 19.0° and 22.4°. The XRD pattern of the physical mixture presents characteristic peaks of both pure compounds, as expected.

The intensities of the peaks recorded in the XRD patterns of the polymer blends depend on the amount of each polymer, since both components crystallize separately [62]. Crystal lattice deformation and crystal size can affect the broadening of XRD peaks, peak intensity, and 2θ-position shifts [63]. Considering this, the drug–polymer XRD patterns suggest that molecular interactions are present in solid cosolvent dispersions, unlike in physical mixtures where these are not established. It is observed that the crystallinity of the system decreases with higher PVP concentration, and, in the case of PLA, the system remains with polymer crystallinity characteristics regardless of the amount of drug added.

#### 3.2.3. Thermogravimetric Analysis (TGA)

**Figure 6:**
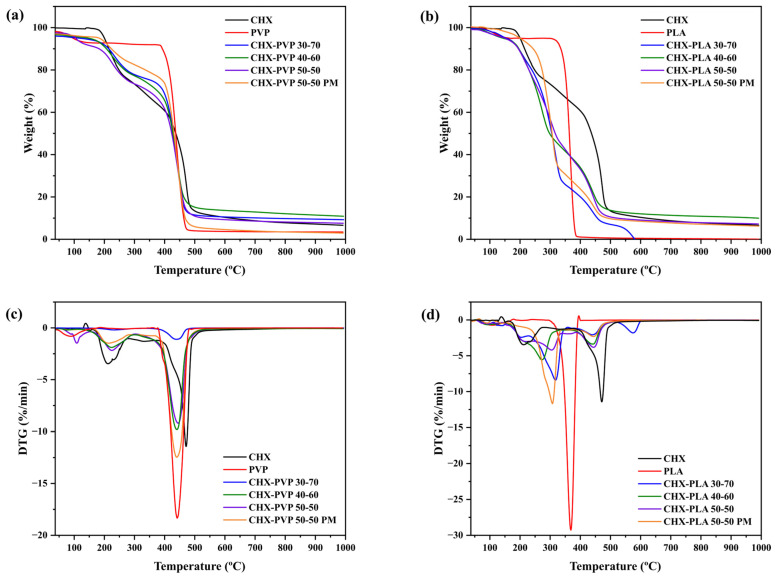
(**a**,**b**) Thermogravimetric and (**c**,**d**) DTG curves of pure compounds, solid dispersions, and physical mixtures (PM) of CHX-PVP and CHX-PLA, respectively.

The thermogravimetric (TG) and derivate TG (DTG) of the solid drug–polymer dispersions and pure compounds are represented in Figure 6; likewise, Table 1 shows the mass loss percentages and the temperatures of the maximum mass loss peaks. The thermogram of chlorhexidine reported two main mass losses at 209.2 °C and 472.7 °C [58,64]. The first loss could be attributed to the degradation point of the biguanide group and the second to the para-chloroaniline group, the same that occurs in the degradation of chlorhexidine [65]. The spectrum of PVP showed a mass loss between 350–480 °C, with a main loss at 437.70 °C, attributed to the cleavage of the carbonyl bond [39], a value that corresponds to TGA analyses reported for PVP, where losses are found around 432 °C [66]. The PLA thermogram presents its highest mass loss between 300–450 °C, with a main loss at 366.2 °C, attributed to chain cleavage and transesterification reactions [39], data that agree with those reported by Frone et al. [67] where the maximum mass loss was reached at 366 °C. In the TGA curves of the solid dispersions made by the CHX-PVP (Figure 6a,c) and CHX-PLA (Figure 6b,d) cosolvent method, it is observed that the maximum mass loss peaks of the samples are mainly found at two temperatures close to the maximum mass loss of the pure components, being between 226.3 ± 4.5 °C and 440.5 ± 2.1 °C for the CHX-PVP systems and between 300.6 ± 19.2 °C and 441.3 ± 1.6 °C for the CHX-PLA systems. On the other hand, the physical mixtures of both dispersions present peaks in the nearby regions where the peaks of the pure CHX, PVP, and PLA compounds appear; however, the endothermic peaks do not reach the same temperatures presented by the cosolvent solid dispersions. Priyadarshini et al. [58], in a study performed with calcium hydroxide (Ca(OH)_2_) microparticles loaded with chlorhexidine (CHX), reported shifts in the decomposition temperature of these microparticles, compared to the pure compounds, proposing the presence of structural changes in the microparticles. Based on the above, the thermograms obtained for the CHX-PVP and CHX-PLA solid powder dispersions suggest the presence of chemical interactions and increase in the stability of the bonds between the components of the systems, due to the change observed between the degradation temperatures of these dispersions compared to the pure compounds; this is observed in the drug–polymer 30-70 dispersions, highlighting an increase in the endothermic peak of CHX, going from 209.2 °C to 231.3 °C (CHX-PVP) and 317.8 °C (CHX-PLA), as well as in the polymer peaks going from 437.7 °C to 438.9 °C for CHX-PVP and from 366.2 °C to 440.8 °C for CHX-PLA. Finally, the thermograms of the physical mixtures propose that in the process of the thermogravimetric analysis the pure compounds could present molecular interactions in the melting stage, increasing the stability of the compounds of the system, increasing the endothermic peak of CHX from 209.2 °C to 216.9 °C (CHX-PVP) and 308.5 °C (CHX-PLA), as well as the peaks of the polymers going from 437.7 °C to 439.1 °C (PVP) and from 366.2 °C to 444.1 °C (PLA).

#### 3.2.4. Differential Scanning Calorimetry (DSC)

**Figure 7:**
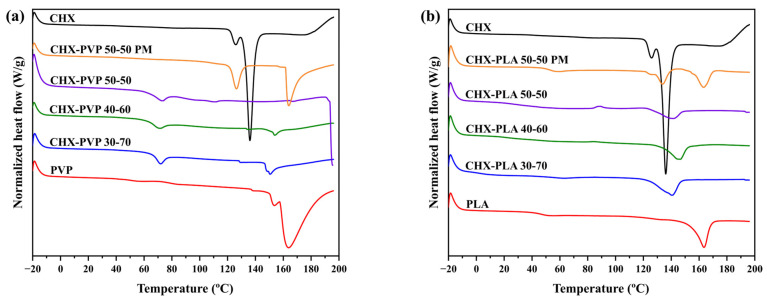
DSC diagrams of pure compounds, solid dispersions, and physical mixtures (PM) of (**a**) CHX-PVP and (**b**) CHX-PLA.

The DSC thermograms of the drug–polymer solid dispersions and pure compounds are shown in Figure 7; on the other hand, their onset, endset, endothermic peak temperature and enthalpy values are shown in Table 2. The DSC thermogram of chlorhexidine shows a characteristic endothermic peak at 136.03 °C corresponding to its melting point [55]. In the spectrum of PVP, two endothermic peaks were identified at 153.06 °C and 163.93 °C corresponding to its melting point (150 °C to 180 °C) [68]. The thermogram of PLA presents an endothermic peak at 163.44 °C being within the range of the values reported for its melting point [69]. In the DSC curves of the solid dispersions made by the CHX-PVP (Figure 7a) and CHX-PLA (Figure 7b) cosolvent method, it is observed that the endothermic peaks of the samples are between the values of the peaks of the pure compounds (136.03–163.93 °C). On the other hand, the physical mixtures of both dispersions present endothermic peaks in the regions where the peaks of the pure compounds CHX, PVP, and PLA appear. Hammannavar et al. [70] state that an endothermic series with lower melting temperatures than the pure compounds suggest the formation of molecular interactions, as well as the presence of crystalline lamellae of different thicknesses. Priyadarshini et al. [58], in a study performed with CHX and calcium hydroxide (Ca(OH)_2_), reported that the absence of detectable CHX domains in the spectra of CHX:Ca(OH)_2_ formulations indicate uniform CHX dispersion. Therefore, the shift of the endothermic peaks of the microparticles indicates the successful incorporation of CHX into the formulations. According to this, the DSC thermograms suggest the presence of molecular interactions in the cosolvent solid dispersions, just as the presence of these interactions in the physical mixtures of these systems is excluded.

### 3.3. Characterization of PVP and PLA Fibers

#### 3.3.1. Morphological Analysis

**Figure 8:**
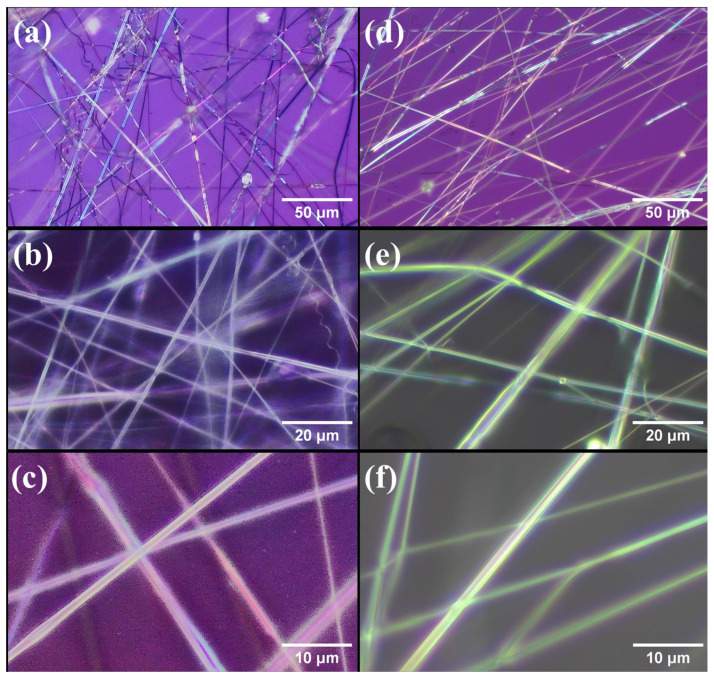
Morphology of fibers obtained by SBS of (**a**–**c**) CHX-PVP and (**d**–**f**) CHX-PLA at magnifications of ×20, ×50, and ×100, respectively. CHX 0.46 *w*/*w*; polymer 6% *w*/*w*.

**Figure 9:**
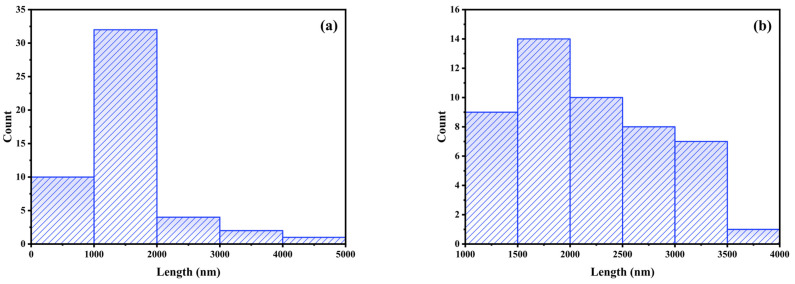
Distribution of the mean diameter of fibers obtained by SBS of (**a**) CHX-PVP and (**b**) CHX-PLA. CHX 0.46 *w*/*w*; polymer 6% *w*/*w*.

Figure 8 shows the appearance of the fibers obtained by SBS of CHX-PVP and CHX-PLA with 6% *w*/*v* polymer concentration and 0.506 mg of chlorhexidine (0.46% *w*/*w*) at magnifications of x20, x50 and x100. Figure 9 shows the histograms corresponding to the distribution of the fiber diameters of CHX-PVP (Figure 9a) and CHX-PLA (Figure 9b). The fibers obtained from the CHX-PVP system present breaks, nonlinearity, and little bead formation. The fibers present diameters that are between 586 and 4099 nm, with a higher frequency in the 1000–2000 nm range and an approximate average diameter of 1625 ± 978 nm. It is also observed that the fibers of the CHX-PLA system present linearity, little bead formation, and do not present breaks. The fibers present diameters that are between 1314 and 3769 nm, with a higher frequency in the 1500–2000 nm range and an approximate average diameter of 2237 ± 760 nm. CHX-PLA fibers showed a higher diameter and greater size dispersion compared to CHX-PVP fibers in which a higher concentration of fibers is observed to be between 1000 and 2000 nm.

#### 3.3.2. Chlorhexidine Release Assays from Solid Dispersions

**Figure 10:**
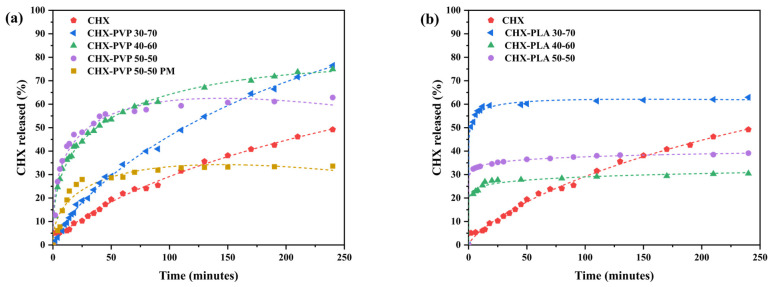
Time-dependent release plots of chlorhexidine from pure CHX, solid dispersions, and physical mixtures (PM) of (**a**) CHX-PVP and (**b**) CHX-PLA with particle size between 160 and 250 µm at 35 °C in water.

Figure 10 shows the release profiles of chlorhexidine with respect to the initial amount present in the samples (7 mg CHX) from the CHX-PVP and CHX-PLA systems with particle size (ps) between 160 and 250 μm (Figure 10a,b) as a function of time at 35 °C (estimated skin surface temperature). In the release profiles of the CHX-PVP systems, it is observed that the release of CHX from the physical mixture is accelerated compared to that of the drug alone (*burst*), 30% is released in about 30 min and stagnates, whereas the drug alone, after a latency period of 20 min, remains dissolving in the medium, slowly and gradually up to 45%. It is possible that this is due to the influence of dissolution interactions between the drug and the polymer which, in some way, facilitate the dissolution of the drug and maintain the equilibrium between the solid and dissolved form. As for the release from the 50–50 and 40–60 solid dispersions, very similar profiles are obtained and they present higher velocity than 30–70, the percentage of CHX released being higher in the last two systems (70%) while with 50–50 it is 10% lower. If the release profiles are compared according to the total amount of drug released (M_t_/M_∞_), CHX and CHX-PVP 30–70 are identical and similar kinetic parameters are obtained, as can be seen in Table 3. Moreover, in the case of CHX-PVP 50–50, whether solid dispersion or physical mixture, the curves overlap, which may indicate that it is the interactions in solution that influence the release, as mentioned above. The profile in the case of the 40–60 dispersion is practically parallel with a difference of 10% below. It can be concluded that the PVP polymer favors the release of CHX and, in particular, in the form of solid dispersion. In the case of PLA, the releases are in general fast, in 30 min the maximum percentage of CHX released has been reached, being the one with the best results the 30–70 solid dispersion (60% released/initial).

Table 3 shows the kinetic parameters obtained by fitting the release data to the mathematical models defined in Section 2.5. Specifically, in all cases, the results corresponding to 80% of CHX released were adjusted with respect to the total amount of drug released in each experiment with the intention of elucidating the predominant kinetic mechanism in each process. It should be recalled that Figure 10 represents the percentage with respect to the initial amount of drug in the samples, being always 7 mg of CHX. As can be seen in Table 3, the releases from CHX-PVP and CHX-PLA solid dispersions and 50–50 physical mixture fit satisfactorily to the Makoid–Banakar and Peppas–Sahlin models. The best fits are indicated in bold in the table. The Makoid–Banakar model is empirical, has no kinetic foundation, and has some shortcomings; it describes the dissolution kinetic properties but does not serve to characterize them adequately. It can be easily deduced that, when the value of c is close to zero, the model agrees with the Korsmeyer–Peppas model and the values of n are similar. The results of k and n or m (as the case may be) obtained for the pure CHX solution and the CHX-PVP 30–70 system are very similar, which logically agrees with the similarity of the profiles already discussed above. The value of c calculated with the Makoid–Banakar model differs in both systems. In the case of CHX alone it is practically zero; however, it presents a value of 0.12 in the solid dispersion, which indicates a clear influence of the presence of the polymer (70%) [44]. As previously commented, the profiles of the CHX-PVP 40–60 and 50–50 dispersions, and the PM 50–50 are superimposable, as reflected in the k values obtained by Korsmeyer-Peppas.

#### 3.3.3. Chlorhexidine Release Assays from Fiber Discs

**Figure 11:**
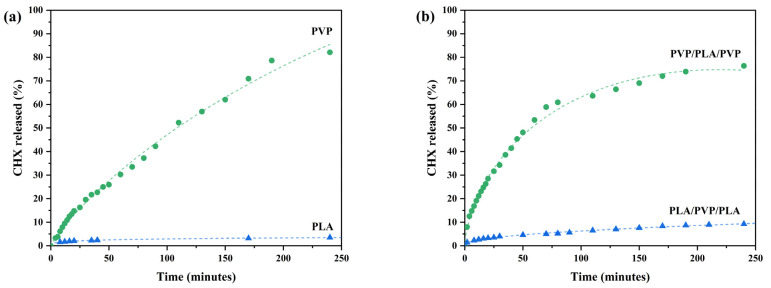
Chlorhexidine release profiles as a function of time from fibers obtained by SBS (**a**) from a single polymer and (**b**) from a mixture of polymers, in water at room temperature.

Discs of PVP and PLA fibers loaded with active principles were set to release in deionized water at room temperature. Mixed disks with fibers of both polymers were also prepared. In Figure 11, the CHX dissolution profiles can be observed from single polymer fiber discs and from the system where PVP fibers surround PLA fibers (PVP/PLA/PVP) and vice versa (PLA/PVP/PLA). It can be observed that, in 4 h, PVP fibers presented a significantly faster drug release compared to PLA fibers (Figure 11a). After observing that the PLA fibers released minimal amounts of drug (3.5%) in 4 h, an aliquot was taken 17 h after starting the releases, observing that the system continued to release the drug, obtaining a final release of 4.4%. This is due to the fact that PVP is hydrophilic; therefore, the system swells and PVP dissolves, contributing to the in vitro release of CHX [71], while PLA fibers, not being water soluble, prove to be stable carriers for the controlled release of chlorhexidine [72]. The release profiles of PVP and PLA fibers satisfactorily fit the Makoid–Banakar and Peppas–Sahlin models with R^2^ coefficient values of 0.995–0.999 (Table 4). The release of CHX from PVP and PLA fibers is initially driven by diffusion mechanisms [46]; considering the obtained c values, it appears to be a zero-order mechanism continued by a first-order process [47]. Likewise, the release profile data for PVP/PLA/PVP and PLA/PVP/PLA fibers also fit well to the Makoid–Banakar and Peppas–Sahlin models with R^2^ of 0.995–0.997. The n values of k and n determined by Korsmeyer–Peppas agree well, in general, with those obtained by these models.

## 4. Conclusions

In this work, the influence of the interactions between the drug CHX and the polymers PVP and PLA in solution and in solid state on the release capacity of the prepared polymeric fibers has been observed. Changes in UV-Vis and fluorescence spectra indicate the presence of molecular interactions between chlorhexidine and the polymers in solutions. FTIR, XRD, and thermal methods analyses show that the drug–polymer powder solid dispersions present molecular interactions that give stability to the system, not so in the physical mixtures. The preparation of solid dispersions by the cosolvent method for the CHX-PVP and CHX-PLA systems facilitates and modulates drug release at 35 °C in water. In the case of the CHX-PVP 50-50 system, up to 85% of the initial drug is released. Releases from PLA are generally fast, reaching, in 30 min, the maximum percentage of CHX released, with the 30-70 solid dispersion being the one with the best results (60%). Particle size control is important, since it has a decisive influence on the speed of chlorhexidine release; an increase in surface area facilitates drug dissolution.

On the other hand, CHX-PVP fibers obtained by Solution Blow Spinning are thinner, and show more breaks and discontinuity in size than CHX-PLA fibers.

The release of CHX from PVP and PVP/PLA/PVP fibers is significantly faster than from PLA and PLA/PVP/PLA fibers, due to the hydrophilic character of PVP.

The release profiles of CHX from the prepared systems, both powder and fibers, satisfactorily fit the Makoid–Banakar and Peppas–Sahlin models with very acceptable R^2^ coefficient values. This implies that the release processes are diffusion driven and suggests that several zero-order type mechanisms are summed followed by the influence of an order 1 mechanism. The Peppas–Sahlin model further suggests that relaxation of the polymer chains facilitates drug release.

## Figures and Tables

**Table 1:** Mass loss and maximum peak temperature obtained from TGA analysis of drug–polymer solid dispersions, physical mixtures, and pure compounds.

Compound/System		Mass Loss (%)	Maximum Peak Temperature (°C)
CHX		27.15	209.2
		52.35	472.7
PVP		5.317 88.10	87.40 437.7
CHX:PVP	30:70	17.43	231.3
		63.84	438.9
	40:60	18.12	222.8
		58.03	439.7
	50:50	6.199	108.0
		18.48	224.7
		62.04	442.8
	50:50 PM	13.98	216.9
		71.71	439.1
PLA		4.739 94.28	108.2 366.2
CHX:PLA	30:70	72.12	317.8
		16.01	440.8
		9.061	575.3
	40:60	4.581	88.96
		51.82	279.9
		25.52	439.9
	50:50	51.68	304.1
		30.36	443.0
	50:50 PM	63.57	308.5
		15.32	444.1

**Table 2:** Onset, endset, peak temperature, and enthalpy obtained from DSC analysis of drug–polymer solid dispersions and pure compounds.

Compound/System		Onset (°C)	Endset (°C)	Peak Temperature (°C)	Enthalpy (J/g)
CHX		132.2	143.3	136.0	94.26
PVP		156.9	190.3	163.9	159.8
		150.6	157.2	153.1	4.249
CHX:PVP	30:70	66.58	67.68	71.83	10.27
		146.4	147.6	150.5	23.96
	40:60	70.01	71.72	75.34	10.38
		147.7	148.5	150.1	7.910
	50:50	63.93	77.62	72.87	8.280
	50:50 PM	121.7	122.3	126.4	22.50
		161.7	162.1	164.0	45.96
PLA		154.8	168.1	163.4	30.30
CHX:PLA	30:70	123.6	146.7	140.4	26.82
	40:60	133.7	151.2	146.3	19.88
	50:50	126.1	147.8	140.3	14.87
	50:50 PM	128.0	138.2	133.7	13.36
		155.6	169.0	163.2	18.58

**Table 3:** Kinetic parameters of CHX release from solid dispersions and physical mixtures with PVP and PLA in water at 35 °C (ps = 160–250 μm).

Polymer Drug:Polumer		Korsmeyer–Peppas			Higuchi		Zero Order		First Order		Makoid–Banakar				Peppas–Sahlin			
		*k_KP_ × 10* ^2^	*n*	*R* ^2^	*k_H_ × 10* ^2^	*R* ^2^	*k*_0_ * × 10* ^2^	*R* ^2^	*k*_1_ * × 10* ^2^	*R* ^2^	*k_MB_ × 10* ^2^	*n*	*c × 10* ^2^	*R* ^2^	*k_D_ × 10* ^2^	*k_R_*	*m*	*R^2^*
**CHX**		2.96 ± 0.23	0.56 ± 0.01	0.994	5.99 ± 0.14	0.962	0.50 ± 0.02	0.880	0.98 ± 0.03	0.983	2.27 ± 0.38	0.73 ± 0.05	0.08 ± 0.04	**0.994**	2.21 ± 0.36	-	0.74 ± 0.04	**0.994**
**PVP**	**30:70**	3.09 ± 0.18	0.64 ± 0.01	0.996	5.98 ± 0.14	0.964	0.50 ± 0.02	0.875	1.02 ± 0.03	0.990	2.18 ± 0.20	0.75 ± 0.03	0.12 ± 0.03	**0.998**	2.17 ± 0.18	-	0.76 ± 0.02	**0.998**
	**40:60**	24.20 ± 1.30	0.27 ± 0.01	0.970	8.34 ± 0.41	0.574	0.62 ± 0.08	-	3.61 ± 0.32	0.815	19.40 ± 1.20	0.35 ± 0.02	0.13 ± 0.03	**0.987**	20.40 ± 1.00	-	0.39 ± 0.02	**0993**
	**50:50**	36.00 ± 3.80	0.21 ± 0.02	0.872	9.41 ± 0.84	0.043	0.67 ± 0.13	-	8.95 ± 0.82	0.918	26.90 ± 2.80	0.34 ± 0.03	0.26 ± 0.05	**0.955**	29.90 ± 2.70	-	0.38 ± 0.03	**0.971**
	**50:50 PM**	28.70 ± 4.80	0.25 ± 0.04	0.848	9.02 ± 0.67	0.488	0.67 ± 0.11	-	6.62 ± 0.46	0.965	18.1 ± 3.7	0.44 ± 0.07	0.33 ± 0.09	**0.928**	19.60 ± 3.70	-	0.47 ± 0.05	**0.931**
**PLA**	**30:70**	82.20 ± 1.40	0.04 ± 0.01	0.992	9.57 ± 1.72	-	0.60 ± 0.18	-	63.20 ± 9.40	0.940	79.40 ± 1.60	0.06 ± 0.01	0.04 ± 0.01	**0.995**	105.00 ± 3.00	-	0.13 ± 0.01	**0.997**
	**40:60**	69.20 ± 2.10	0.07 ± 0.01	0.984	9.23 ± 1.36	-	0.60 ± 0.16	-	22.00 ± 2.40	0.920	64.20 ± 2.70	0.10 ± 0.02	0.06 ± 0.03	**0.989**	75.00 ± 4.50	-	0.18 ± 0.02	**0.992**
	**50:50**	77.00 ± 0.40	0.05 ± 0.01	0.999	9.66 ± 1.32	-	0.65 ± 0.16	-	32.60 ± 4.60	0.914	76.60 ± 0.50	0.05 ± 0.01	0.01 ± 0.01	**0.999**	89.30 ± 1.30	-	0.06 ± 0.02	**0.999**

(-) indicates negative values.

**Table 4:** Kinetic parameters of CHX release from single and mixed polymer fibers in water at room temperature.

Polymer/System	Korsmeyer–Peppas			Higuchi		Zero Order		First Order		Makoid–Banakar				Peppas–Sahlin			
	*k_KP_*	*n*	*R* ^2^	*k_H_*	*R* ^2^	*k*_0_	*R* ^2^	*k*_1_	*R* ^2^	*k_MB_*	*n*	*c x 10* ^2^	*R* ^2^	*k_D_*	*k_R_*	*m*	*R^2^*
**PVP**	1.84 ± 0.14	0.74 ± 0.02	0.994	5.71 ± 0.21	0.918	0.51 ± 0.02	0.940	0.91 ± 0.03	0.974	1.36 ± 0.22	0.83 ± 0.04	0.09 ± 0.04	**0.995**	1.31 ± 0.19	-	0.85 ± 0.04	**0.995**
**PLA**	24.5 ± 0.80	0.20 ± 0.01	0.996	4.17 ± 0.63	0.139	0.12 ± 0.04	-	2.64 ± 0.54	0.705	22.40 ± 0.70	0.23 ± 0.01	0.01 ± 0.01	**0.999**	22.30 ± 0.80	-	0.27 ± 0.01	**0.999**
**PVP/PLA/PVP**	11.20 ± 0.90	0.42 ± 0.02	0.972	7.70 ± 0.17	0.949	0.62 ± 0.05	0.319	2.10 ± 0.07	0.978	6.18 ± 0.42	0.62 ± 0.02	0.27 ± 0.02	**0.996**	6.34 ± 0.38	-	0.64 ± 0.02	**0995**
**PLA/PVP/PLA**	3.90 ± 0.18	0.46 ± 0.01	0.995	3.19 ± 0.04	0.990	0.12 ± 0.02	0.258	0.35 ± 0.02	0.898	4.72 ± 0.35	0.42 ± 0.02	-	**0.997**	4.59 ± 1.54	0.68 ± 1.51	0.32 ± 0.10	**0.997**

(-) indicates negative values.

## Data Availability

The original contributions presented in this study are included in the article. Further inquiries can be directed to the corresponding author.

## Abbreviations

The following abbreviations are used in this manuscript:

PVP	Polyvinylpyrrolidone
PLA	Polylactic acid
CHX	Chlorhexidine
PM	Physical mixture
SD	Solid dispersions
SBS	Solution Blow Spinning
PVA	Polyvinyl alcohol
PCL	Polycaprolactone
ES	Electrospinning
UV-Vis	Ultraviolet Visible Spectroscopy
FTIR	Fourier Transform Infrared Spectroscopy
XRD	X-Ray Diffraction
TGA	Thermogravimetric Analysis
DSC	Differential Scanning Calorimetry
